# Primary hepatic angiosarcoma with noncirrhotic portal hypertension: A case report

**DOI:** 10.3389/fonc.2023.1037820

**Published:** 2023-02-02

**Authors:** Xuwei Wu, Xia Yu, Qiaorong Gan, Bin Wang, Zhaowang Lin, Yu Shi, Zuxiong Huang

**Affiliations:** ^1^ Department of Hepatology, Mengchao Hepatobiliary Hospital of Fujian Medical University, Fuzhou, China; ^2^ State Key Laboratory for Diagnosis and Treatment of Infectious Diseases, National Clinical Research Center for Infectious Diseases, Collaborative Innovation Center for Diagnosis and Treatment of Infectious Diseases, The First Affiliated Hospital, College of Medicine, Zhejiang University, Hangzhou, China; ^3^ Department of Pathology, Mengchao Hepatobiliary Hospital of Fujian Medical University, Fuzhou, China; ^4^ Department of Imaging, Mengchao Hepatobiliary Hospital, Fujian Medical University, Fuzhou, China

**Keywords:** primary hepatic angiosarcoma, noncirrhotic portal hypertension, diagnosis, treatment, pathology

## Abstract

**Background:**

Primary hepatic angiosarcoma (PHA) is a rare malignant tumor of mesothelial tissue origin in the liver. The diagnosis of PHA relies on pathology, and it is frequently misdiagnosed as multiple hepatic hemangioma. Noncirrhotic portal hypertension is a relatively rare pathological manifestation, and there are few reports of PHA as an uncommon cause of noncirrhotic portal hypertension.

**Case summary:**

A 36-year-old male was admitted with abnormal liver function and suspected drug-induced liver injury (DILI), initially manifesting as multifocal hepatic hemangioma. The liver biopsy revealed features of noncirrhotic portal hypertension (NCPH), and the patient was eventually diagnosed with multifocal hepatic angiosarcoma.

**Conclusion:**

Patients with PHA may present with NCPH in the liver due to injury to hepatic sinusoids; therefore, it is necessary to consider the possibility of unsampled vascular malignancy when hepatic masses are identified, and the histology is consistent with PHA.

## Introduction

Primary hepatic angiosarcoma (PHA), also known as hepatic vascular endothelial sarcoma, hepatic malignant angioendothelioma, or Kupffer cell sarcoma, is a rare malignant tumor of mesothelial tissue origin, accounting for 0.1–2% of primary liver malignancies (1, 2). It is the most common primary malignant mesothelial tumor in the liver and was first reported by Block in 1974 (3). PHA occurs mostly in adults, especially those aged 50 to 70 years old, with a male-to-female ratio of 4:1. Potential pathogenic factors include exposure to chemicals such as arsenic, vinyl chloride monomer, thorium dioxide, and radium (4). The clinical signs and symptoms of primary hepatic angiosarcoma are similar to those of chronic liver diseases, and patients often present with abdominal pain, weight loss, fatigue, and anorexia. Hepatosplenomegaly, abdominal effusion, and jaundice are also common in elderly male patients (5). PHA is fatal, with most patients dying within 6 months of liver failure or bleeding (2).

Non-cirrhotic portal hypertension (NCPH) is a heterogeneous group of liver diseases of vascular origin, usually manifesting as portal hypertension (PHT) but with preserved hepatic synthetic function and near-normal hepatic venous pressure gradient (HVPG) (6). PHA is a rare cause of NCPH; thus, case reports of PHA combined with NCPH are very rare.

Herein, we present the case of a young male patient with an abnormal liver function who was eventually diagnosed with hepatic angiosarcoma. The patient was hospitalized twice, initially misdiagnosed as drug-induced liver injury complicated by NCPH and hepatic hemangioma, and was finally diagnosed as liver angiosarcoma.

## Case presentation

**Figure 1:**
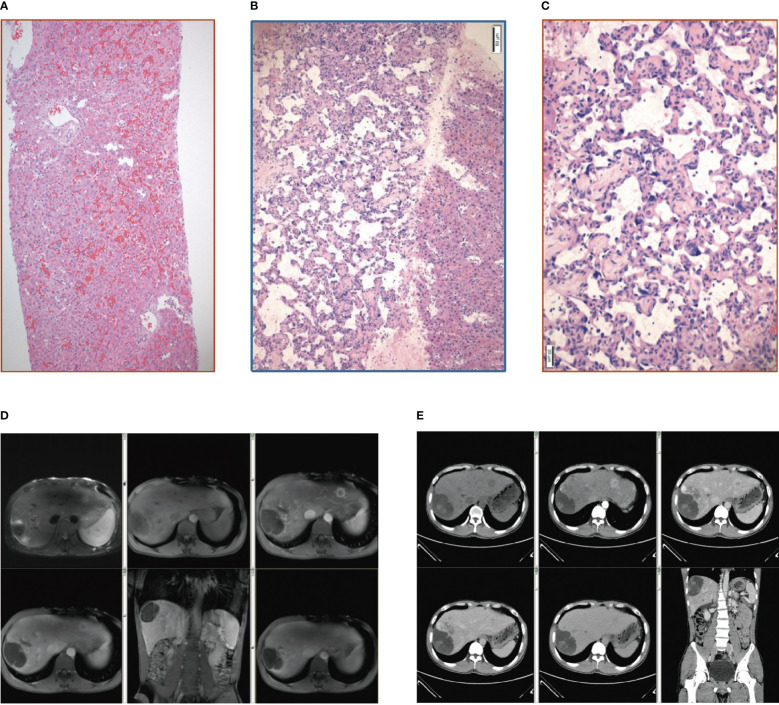
**(A)** Pathology of the liver tissue: HE staining (X10). **(B)** Pathology of the liver tissue: HE staining (X100). **(C)** Pathology of the liver tissue: HE staining (X200). **(D)** Contrast-enhanced MRI scan of the upper abdomen. **(E)** Whole abdominal CTV.

A 36-year-old man with a 20-year history of nephrotic syndrome with normal renal function and fluctuating urine protein levels had been treated with glucocorticoids and traditional Chinese medicine for more than 10 years. He was first hospitalized due to abnormal liver function. Routine laboratory tests revealed mild abnormal liver function [total bilirubin (TBIL) 41.9 µmol/L, direct bilirubin (DBIL) 22.7 µmol/L, alanine aminotransferase (ALT) 21.6 U/L, aspartate aminotransferase (AST) 81.1 U/L, gamma-glutamyl transferase (GGT) 517.5 U/L, alkaline phosphatase (ALP) 323 U/L]. Doppler ultrasound, CT scan, and contrast-enhanced MRI scan of the upper abdomen demonstrated multiple small masses in the liver, so hemangioma was considered. Also, the enlarged spleen and collateral vessels indicated the presence of portal hypertension. The patient underwent a percutaneous liver biopsy and soon after felt pain and discomfort in the right upper abdomen with transient blood pressure decline. The ultrasound confirmed bleeding and hemorrhagic shock due to the liver biopsy. The patient recovered after fluid rehydration, blood transfusion, and other supportive treatments. Pathological examination of the liver biopsy (**Figure 1A**) revealed congested hepatic sinusoids and peri-sinusoid fibrosis, as well as dilation of the interlobular veins and some extended into the surrounding hepatic sinus. These changes in liver histology were consistent with NCPH features that represent venous outflow stenosis with portal hypertension. There was no evidence of extra-hepatic portal vein obstruction, and the patient was diagnosed with a drug-induced liver injury [the Roussel Uclaf Causality Assessment Method (RUCAM) score 5] and secondary NCPH. Ursodeoxycholic acid was given, and regular outpatient follow-up was performed.

Three months after discharge, an outpatient contrast-enhanced MRI scan of the upper abdomen depicted enlarged hepatic masses. Considering the possibility of malignancy (**Figure 1D**), the patient was readmitted to the hospital. Routine laboratory tests revealed abnormal liver function [albumin (ALB) 21 g/L, TBIL 35.7 µmol/L, DBIL 14.0 µmol/L, ALT 67 U/L, AST 70 U/L, GGT 147 U/L, ALP 240 U/L]. Liver contrast-enhanced ultrasound and positron emission tomography-computed tomography (PET-CT) indicated the possibility of primary liver malignancy with intratumoral hemorrhage. Whole abdominal CTV demonstrated multiple masses in the liver and an enlarged spleen with a spleen-renal venous shunt (**Figure 1E**). The patient was transferred to the hepatobiliary surgery department and underwent laparoscopic resection of the liver lesions. Liver lesion biopsy pathology (**Figures 1B, C**) demonstrated that the tumor cells were slit and cable-like with a vascular network and growth along the liver sinusoids. Immunohistochemistry revealed high expression of CD34, CD31, and ERG (ETS transcription factor).

The patient was diagnosed with primary hepatic angiosarcoma and was treated with lenvatinib. In May 2020, he stopped taking lenvatinib due to progressively exacerbating liver function and died of severe intra-abdominal infection and acute kidney failure.

## Discussion

PHA is a rare malignant tumor originating from the liver sinusoidal endothelial cells. Herein, we reported the case of a male patient who was admitted to hospital due to abnormal liver function. CT and MRI play an important role in diagnosing hepatic angiosarcoma, and there are four main types of PHA radiological presentation: multiple nodules, massive masses, massive masses with multiple nodules, and diffuse invasive micronodular tumors. Most lesions present hypodense on CT scans, but some are hyperdense due to spontaneous intraperitoneal or intratumoral hemorrhage (7). On contrast injection, most nodular lesions depict low-density enhancement, and some show irregular or annular enhancement. The MRI reflects the hemorrhagic, heterogeneous, and multivascular nature of the PHA lesions, typically areas of high signal intensity on T1-weighted images and distinct heterostructures on T2-weighted images, suggesting intratumoral hemorrhage and fibrous septa (8). Hepatic angiosarcoma can be distinguished from hepatic hemangiomas or other liver tumors by CT and MRI. Previous studies have reported that using non-enhanced, multiphase-enhanced, and late-delayed CT and MR imaging is an optimal imaging technique for accurately assessing PHA (7, 9). However, due to its rarity, PHA is easily misdiagnosed as multiple hepatic hemangioma.

The typical histology of hepatic angiosarcoma is tumor vascular-like cavities lined by spindle cells that project into the lumen to form papillary structures. The degree of differentiation of tumor cells varies greatly, with well-differentiated tumor cells resembling hemangioma and poorly differentiated tumor cells having obvious atypia. Tumor cells can look spongy with no obvious vascular space, giant cells, and pathological mitoses. They often spread along the sinus, terminal liver vein, and portal vein branches, and multilayer or prominent vascular lumen growth on the liver plate causes liver plate dissociation. Hepatocytes atrophy or disappear, the vascular cavity is enlarged, and the rapidly growing tumor tissue has visible residual hepatocytes, vascular lumen visible blood clots, and tumor cell debris (10). Immunohistochemically, liver sections are positive for CD31, CD34, D2-40, and factor VIII-associated antigens, with CD31 and factor VIII-associated antigens being the most specific markers (11).

Interestingly, this patient also presented with NCPH features. NCPH is a vascular liver disease of unknown etiology that presents clinically as portal hypertension (examples include thrombocytopenia secondary to hypersplenism and bleeding from esophagogastric varices or hypertensive gastropathy) with preserved hepatic synthetic function and near-normal hepatic venous pressure gradient (HVPG). It is characterized by increased pressure in the portal vein and its branches due to fibrosis of the intima and the absence of cirrhosis. The diagnosis of NCPH is mainly based on the following signs: a) hepatic hemodynamics and portal venography showing a high gradient of the portal sinus with fewer branches and increased portal caliber in the absence of thrombosis; b) liver biopsy showing thickening of portal vein fibers and no evidence of cirrhosis, necrosis, or inflammation (12). Although there is evidence that certain toxic substances (e.g., arsenic salts, thorium sulfate, vinyl chloride) may cause NCPH, the cause is often not identified. It has been suggested that some histological lesions of NCPH, such as hepatic sinusoidal cell hyperplasia, may lead to the development of angiosarcoma (4, 13, 14); however, there is no clear association between NCPH and hepatic angiosarcoma. In addition, a series of specific risk factors, such as arsenic, vinyl chloride, and androgenic steroids, which have a latency period of up to 20 years for their carcinogenic effects, have been reported in 40% of patients with hepatic angiosarcoma (15, 16). As previously mentioned, these risk factors are also associated with NCPH development; therefore, NCPH and hepatic angiosarcoma may share the same triggering environmental factors. To our knowledge, there is a lack of literature describing the association between hepatic angiosarcoma and NCPH, with only eight published reports of NCPH and hepatic angiosarcoma-related cases, of which two patients had prior exposure to arsenic (17, 18), four patients with prior exposure to polyvinyl chloride (19), and two patients with unknown prior exposure history (19, 20). Furthermore, there have been reported cases of liver failure (21), which may be caused by portal fibrosis, destruction of the terminal portal nerve root in the liver, and liver parenchyma atrophy secondary to poor portal perfusion. In addition, PHA liver biopsies are extremely prone to bleeding, and as in this case, the liver biopsy caused transient hemorrhagic shock. Therefore, in patients with suspected PHA, non-surgical liver biopsy should be performed with extreme caution and careful evaluation before surgery is required.

Due to the rare incidence of PHA, there are no established treatment guidelines. Hepatectomy may be considered when the lesion is localized to one liver lobe. Patient survival may be prolonged with improved diagnostic techniques and liver transplantation, but the recurrence rate remains very high, and the prognosis is very poor (10). Also, PHA is considered an absolute contraindication to liver transplantation due to its dismal results. There are a few reports that chemotherapy is effective for PHA, and palliative care can be considered in cases of inoperable surgery. Local chemotherapy, such as transcatheter arterial chemoembolization (TACE) or systemic chemotherapy, can effectively prolong the life of patients, even equal to surgical outcomes (22, 23). PHA is a vascular-derived malignancy, so anti-angiogenic therapy may be a new potential strategy. There is a report of thalidomide combined with radiotherapy in a PHA patient with neck metastasis achieving a tumor-free state (23). Targeted therapies such as sorafenib and bevacizumab have limited efficacy in treating primary angiosarcoma of other organs; however, only one case of PHA was included in these studies (24, 25). An attempt to combine pazopanib, a PD-1 inhibitor, and RAK cells yielded effective results in an elderly PHA patient (26). These new approaches, alone or in combination with other therapeutic modalities, such as surgery and chemotherapy, need further investigation to assess their role in prolonging patient survival. Personalized therapeutic algorithms according to the genetics, molecular biology, histopathological features, and behavior of the tumors should be elaborated for the management of PHA patients.

## Data availability statement

The raw data supporting the conclusions of this article will be made available by the authors, without undue reservation.

## Ethics statement

Written informed consent was obtained from the individual(s) for the publication of any potentially identifiable images or data included in this article.

## Author contributions

Author contributions: XW, XY, QG, BW, ZL collected and analyzed the data. XW, XY wrote the paper. YS and ZH critically reviewed the manuscript. All authors contributed to the article and approved the submitted version.
